# Evidence for the involvement of lung-specific γδ T cell subsets in local responses to *Streptococcus pneumoniae* infection

**DOI:** 10.1002/eji.200737216

**Published:** 2007-12

**Authors:** Alun C Kirby, Darren J Newton, Simon R Carding, Paul M Kaye

**Affiliations:** 1 Immunology and Infection Unit, Hull York Medical School and Department of Biology, University of YorkYork, UK; 2 Research Institute for Molecular and Cellular Biology, University of LeedsLeeds, UK

**Keywords:** Cell homing, γδ T cells, Lung inflammation, Mucosal immunity

## Abstract

Although γδ T cells are involved in the response to many pathogens, the dynamics and heterogeneity of the local γδ T cell response remains poorly defined. We recently identified γδ T cells as regulators of macrophages and dendritic cells during the resolution of *Streptococcus pneumoniae*-mediated lung inflammation. Here, using PCR, spectratype analysis and flow cytometry, we show that multiple γδ T cell subsets, including those bearing Vγ1, Vγ4 and Vγ6 TCR, increase in number in the lungs of infected mice, but not in associated lymphoid tissue. These γδ T cells displayed signs of activation, as defined by CD69 and CD25 expression. *In vivo* BrdU incorporation suggested that local expansion, rather than recruitment, was the principal mechanism underlying this increase in γδ T cells. This conclusion was supported by the finding that pulmonary γδ T cells, but not αβ T cells, isolated from mice that had resolved infection exhibited lung-homing capacity in both naive and infected recipients. Together, these data provide novel insights into the origins of the heterogeneous γδ T cell response that accompanies lung infection, and the first evidence that inflammation-associated γδ T cells may exhibit distinct tissue-homing potential.

## Introduction

γδ T cells are a rare population of T lymphocytes in most tissues. The γδ TCR^+^ population can be subdivided based on the expression of specific Vγ and Vδ TCR chains [1], defined here using the nomenclature of Heilig and Tonegawa [2]. The resultant clonal and oligoclonal subsets may preferentially associate with specific tissues [3]. For example, expression of the Vγ5 chain is predominantly restricted to dendritic epithelial γδ T cells found in skin [4] and Vγ6^+^ cells are the major reproductive tract γδ T cell population [5]. In contrast, heterogeneous populations of γδ T cells are found in other tissues, including the lung.

Lung γδ T cells comprise predominantly Vγ1^+^ and Vγ4^+^ subsets, with Vγ6^+^ cells also present [6–8]. These subsets may have distinct functions, often acting as immunoregulatory cells. For example, Vγ1^+^ cells promote airway hyper-responsiveness, whereas Vγ4^+^ cells suppress this activity [9]. In a model of chronic pulmonary fibrosis, canonical Vγ6^+^ cells may down-regulate αβ T cell-mediated pathology [10]. Contrastingly, both Vγ1^+^ and Vγ4^+^ subsets contribute to cytokine production during infection with influenza [11, 12], or following mycobacterial exposure [13]. Immunoregulatory γδ T cell responses associate with a range of pulmonary infections, including *Nocardia asteroides* [14], *Klebsiella pneumoniae* [15] and *Cryptococcus neoformans* [16]. However, in general, the relative contributions of specific subsets to these responses have not been determined.

Substantial evidence exists therefore to place γδ T cells as central immunoregulatory cells not only within the lung but in many tissues [3, 17]. Despite the importance of immunoregulation in generating appropriate immune responses, γδ T cells remain relatively poorly characterized during responses to infection. During resolving *Streptococcus pneumoniae*-induced lung inflammation [18, 19], γδ T cell numbers increase >30-fold and are responsible for regulating macrophage and dendritic cell numbers during the resolution phase of inflammation [20]. Using RT-PCR, spectratyping, flow cytometry and analysis of cell cycle progression, these γδ T cells are shown here to represent an expansion of multiple lung-resident γδ T cell subsets and to have an activated phenotype. The response is lung restricted, and γδ T cells isolated from resolving lungs preferentially home back to the lungs of recipient mice in an inflammation-independent manner. Together, these data provide new insights into the lung γδ T cell population and their tissue-specific nature.

## Results

### Multiple Vγ chain-expressing γδ T cell populations in naive and inflamed lungs

**Figure 1:**
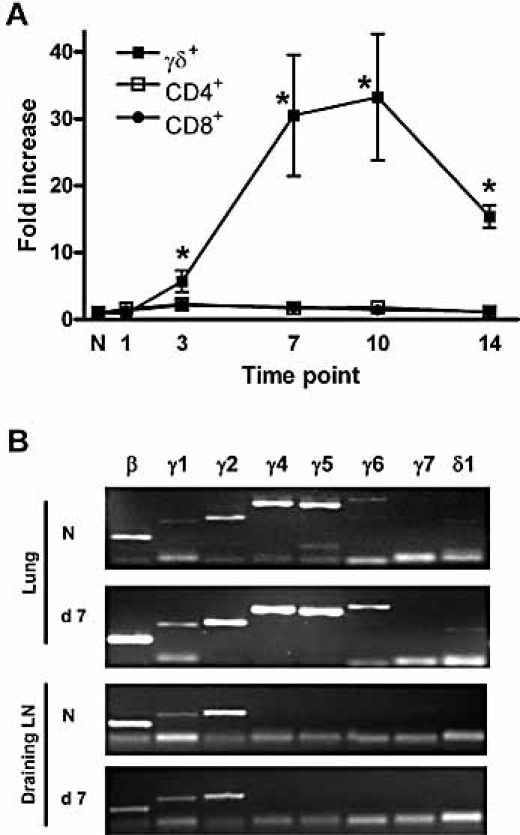
Pulmonary γδ T cells increase following *S. pneumoniae* challenge. (A) Gated CD3^+^TCRβ^+^CD4^+^, CD3^+^TCRβ^+^CD8^+^ and CD3^+^TCRβ^–^TCRδ^+^ populations in the lung were examined in naive mice and at various time points following *S. pneumoniae* challenge. The fold increase in absolute number of each population at days 1–14 following challenge is shown. The graph represents 6–18 mice at each time point. *p <0.05 *vs.* naive control by Student's *t*-test. (B) Purified γδ T cells from naive (N) lung, or from lung at day 7 post *S. pneumoniae* challenge, were analyzed for mRNA encoding various Vγ chains or the Vδ1 chain, with β-actin (β) expression used as a control. Draining LN γδ T cells were analyzed in comparison. Representative samples are shown in each case.

Data characterizing γδ T cells during pathogen-induced responses remain sparse. We have used a model of resolving *S. pneumoniae* infection [18, 19] to examine the pulmonary γδ T cell response following local pathogen-induced inflammation [20]. In this model, intranasal challenge with *S. pneumoniae* serotype 6B had little effect on the numbers of lung CD4^+^ or CD8^+^ T cell populations (Fig. 1A) over the subsequent 14 days. In contrast, the pulmonary γδ T cell population was significantly increased, with a >30-fold increase in γδ T cell number observed at the peak of the response (days 7–10; Fig. 1A). While the γδ T cell response subsequently subsided, it remained significantly elevated at day 14 compared with naive controls (Fig. 1A).

Given this striking change in the lung γδ T cell population, we next addressed whether one or more specific γδ T cell subsets were responding to *S. pneumoniae* challenge. Qualitative PCR analysis of highly purified lung γδ T cells from naive animals (*n* = 4 pools of six mice) showed a mixed population, with mRNA for Vγ1, Vγ2, Vγ4, Vγ5 and Vγ6 detectable (Fig. 1B). Analysis of γδ T cells purified at day 7 post *S. pneumoniae* challenge (*n* = 6 pools of three to six mice) also showed expression of mRNA for Vγ1, Vγ2, Vγ4, Vγ5and Vγ6. The Vγ5 chain commonly associates with Vδ1 in DETC, although expression of mRNA for Vδ1 was barely detectable in γδ T cells purified from naive lung. In contrast, mRNA for Vδ1 was consistently detectable in samples from day 7 post challenge (Fig. 1B). Vγ7 mRNA was not detected in any samples from either naive or infected mice.

In contrast with the lung, sorted γδ T cells from the draining LN of naive (*n* = 3 pools of three to six mice) and *S. pneumoniae*-challenged mice (*n* = 3 pools of three to six mice) expressed only Vγ1, Vγ2 and, weakly, Vγ4 mRNA (Fig. 1B).

### Expansion of Vγ1^+^ and Vγ4^+^ γδ T cells following pneumococcal challenge

**Figure 2:**
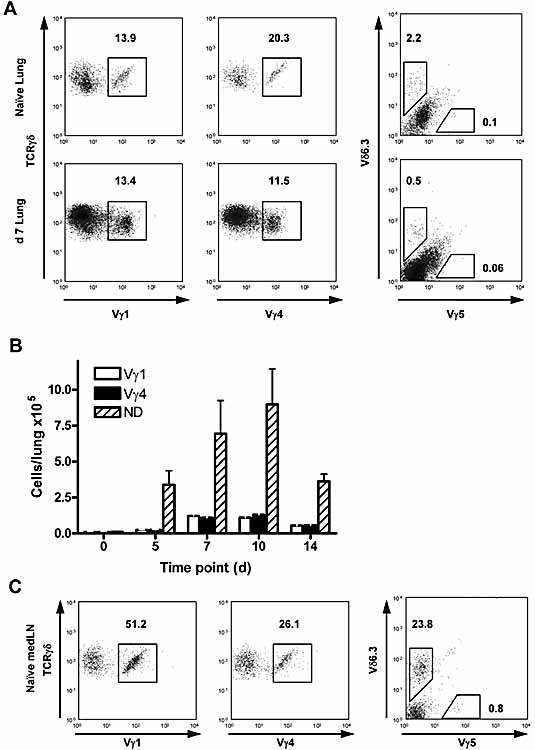
Expression of Vγ/Vδ chains assessed by flow cytometry. (A) Total lung cells from naive mice, or mice challenged 7 days previously with *S. pneumoniae* were stained for the expression of various Vγ chains and of Vδ6.3. Cells shown are gated on size and granularity, and as CD3^+^TCRβ^–^TCRδ^+^ cells. Six to twelve individual mice, or pools of at least three mice (naive only), were analyzed in each case. (B) Absolute numbers of Vγ subsets in naive and *S. pneumoniae*-challenged lungs at various time points. Bars represent mean (± 1 SD) Vγ1 (open bars), Vγ4 (closed bars) or Vγ1^–^Vγ4^–^TCRδ^+^ cells in total lung (ND, not defined: hatched bars). (C) Draining LN cells from naive mice stained as for (A).

To confirm the PCR analysis and to obtain quantitative data regarding γδ T cell subsets, flow cytometric analysis of Vγ1, Vγ4, Vγ5 and Vδ6.3 expression was carried out on total lung cells. In naive mice, Vγ1^+^ cells represented a mean 12.1 ± 2.0% (*n* = 9) of total γδ T cells. At day 7 following *S. pneumoniae* challenge, this proportion was maintained, with 13.5 ± 1.1% (*n* = 12) of γδ T cells expressing Vγ1 (Fig. 2A). Accounting for the increased total viable lung cell number associated with inflammation, a significant increase in the mean number of Vγ1^+^ cells results following pneumococcal challenge. The absolute number of pulmonary Vγ1^+^ cells rose from 3.6 ± 0.6 × 10^3^ in naive mice, to 1.25 ± 0.1 × 10^5^ at day 7 (*p* <0.001; Fig. 2B), a 35-fold increase.

Vγ4^+^ cells were also well represented among γδ T cells in the naive lung (mean 21.5 ± 1.5%) (Fig. 2A). While pneumococcal challenge resulted in a significant decrease in the relative proportion of Vγ4^+^ cells at day 7 (12.3 ± 1.2%; *p* = 0.001), this represented an almost 20-fold increase in their absolute number, from a mean of 6.5 ± 0.4 × 10^3^ cells/lung in naive mice to 1.14 ± 0.1 × 10^5^ at day 7 (*p* <0.001; Fig. 2B).

Expression of the Vδ6.3 chain, which can associate with Vγ1 [21], was consistently restricted to a small proportion of naive lung γδ T cells (mean 1.9 ± 0.3%), which was reduced to 0.3 ± 0.2% at day 7. While this translated to a fourfold increase in the mean number of these cells in the lung, they remained relatively infrequent at day 7 (mean 2.6 ± 1.4 × 10^3^ cells/lung). No distinct Vγ5^+^ population was present in either naive animals or following *S. pneumoniae* challenge, as determined by flow cytometry. The increase in number of both the Vγ1 and Vγ4 subsets, as well as of the remaining undefined γδ T cells, followed similar kinetics (Fig. 2B). That is, in each case, the majority of the increase occurred between days 5 and 7 post challenge and peaked at days 7–10.

In comparative stainings of naive draining LN (Fig. 2C), >75% of γδ T cells expressed either Vγ1 or Vγ4. These results indicate that a significant proportion of lung γδ T cells (>65% in naive and >75% in infection) remain unidentifiable by the current panel of antibodies, which is not due to an inability of the antibodies to recognize their specific TCR in our hands. PCR analysis (Fig. 1B) suggests that these cells may comprise Vγ2^+^ and/or Vγ6^+^ cells, for which antibodies are not available. Nevertheless, together these results clearly demonstrate that *S. pneumoniae* challenge results in increased numbers of multiple γδ T cell subsets in the lung.

### Non-productive Vγ5 transcripts are present in lung γδ T cells

The expression of Vγ5 mRNA in naive and inflamed lungs was unexpected, since Vγ5^+^ T cells are reportedly restricted to skin [4], with minor populations found in murine mammary gland [22] and in spleens of Vγ1^–/–^ mice [23]. However, expression of cell surface Vγ5 protein was not detected by flow cytometry (Fig. 2A). We therefore carried out spectratyping analysis of PCR products to determine whether the Vγ5 transcripts were putatively productive.

**Figure 3:**
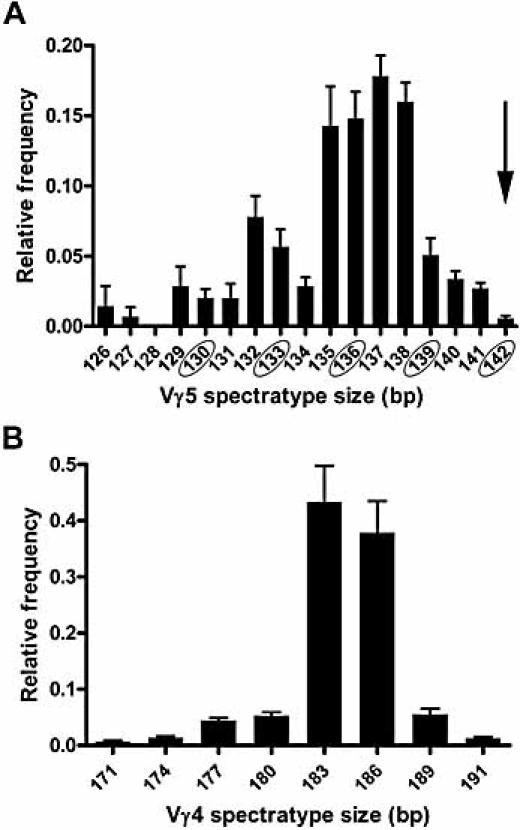
Spectratype analysis of putative pulmonary Vγ5 mRNA transcripts. PCR products from mRNA analysis of lung γδ T cells (Fig. 1B) were excised, purified and spectratyped. The mean relative frequencies (± 1 SEM; *y* axis) of spectratypes (*x* axis) from (A) Vγ5 (*n* = 12) and (B) Vγ4 (*n* = 6) PCR products is shown. (A) The canonical Vγ5 (DETC) spectratype at 142 bp is indicated (arrow) and other putative in-frame spectratypes are circled.

The canonical, oligoclonal dendritic epithelial γδ T cell population has Vγ5 spectratype lengths of 142 and 145 bp [23]. However, Vγ5 transcripts from both naive γδ T cells and γδ T cells from *S. pneumoniae*-challenged mice comprised spectratypes ranging from 126 to 142 bp. The in-frame, 142-bp spectratype was among the least represented of those observed, with no apparent bias towards other putative productive, in-frame transcripts (139, 136, 133 and 130 bp) compared with non-productive (out-of-frame) spectratypes (Fig. 3A). In contrast, analysis of Vγ4 PCR products showed dominant (>90%) expression of the major productive (183 and 186 bp) spectratypes (Fig. 3B). Together, flow cytometry and spectratype data strongly suggest that Vγ5-expressing T cells do not occur in naive lung, and are not induced following *S. pneumoniae* challenge.

### γδ T cells exhibit a highly activated phenotype following *S. pneumoniae* challenge

**Figure 4:**
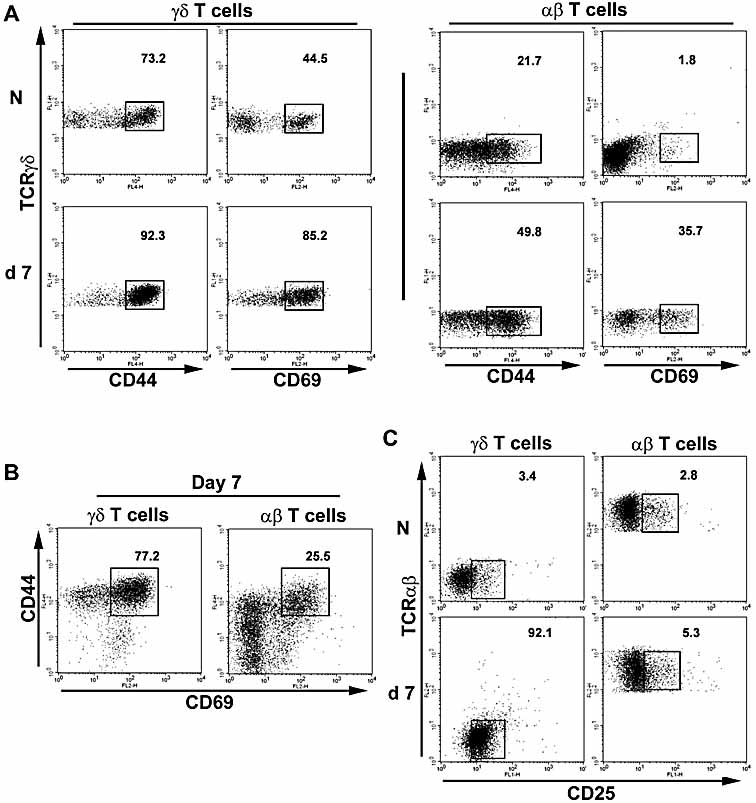
Lung γδ T cells have a highly activated phenotype. Lung γδ (CD3^+^TCRβ^–^TCRδ^+^) or αβ (CD3^+^TCRβ^+^TCRδ^–^) T cells from naive (N; *n* = 6) mice, or mice challenged 7 days previously with *S. pneumoniae* (*n* = 6) were stained for the expression of the activation markers CD44 and CD69. (A) Numbers indicate percentages of CD44^HI^ and CD69^HI^ cells among γδ (left panels) and αβ (right panels) T cells, gated relative to isotype control mAb staining (not shown). (B) Gated γδ (left plot) and αβ (right plot) T cells from day 7 post challenge analyzed for concomitant expression of CD44 and CD69. Gated CD44^HI^CD69^HI^ cells are shown, along with the percentage of CD44^HI^CD69^HI^ cells. (C) Gated γδ (left plots) and αβ (right plots) T cells from naive or day 7 mice analyzed for CD25 expression. Gates indicate CD25^+^ cells. All axes represent log fluorescence intensity and representative plots are shown.

To determine whether lung γδ T cells are activated as a result of *S. pneumoniae* challenge, activation-associated surface marker expression and inflammatory cytokine production were examined by flow cytometry. In naive lungs, significantly more γδ T cells were CD44^HI^ (mean 72.6 ± 5.0%) than were αβ T cells from the same individuals (22.6 ± 1.6%, *p* = 0.001). Similarly, 43.3 ± 4.9% of naive lung γδ T cells expressed the early activation marker CD69, compared with only 2.1 ± 0.3%% of naive αβ T cells (*p* = 0.004; Fig. 4A).

At day 7 following *S. pneumoniae* challenge, 92 ± 3% of γδ T cells were CD44^HI^, significantly increased from naive levels (*p* <0.01.). While CD44^HI^ αβ T cells were also increased compared with naive controls (50.4 ± 1.9%; *p* <0.01), they remained significantly less represented than among γδ T cells at the same time point (*p* <0.001; Fig. 4A). A similar pattern was observed for CD69 expression, where the proportions of both γδ (83 ± 5%) and αβ T cells (36.0 ± 4.1%) expressing CD69 at day 7 post challenge were increased compared to their naive counterparts (*p* <0.01 in both cases). However, CD69 expression among γδ T cells remained more prevalent than among the αβ T cell population in the lung (*p* <0.001; Fig. 4A). At day 7 following challenge, 75 ± 3% of γδ T cells were CD44^+^CD69^+^, in comparison to only 27.2 ± 2.4% of αβ T cells (*p* <0.001; Fig. 4B).

The expression of CD25, a marker of T cell activation *in vitro* while associated with a regulatory T cell phenotype *in vivo* [24], further delineated the two T cell populations. Whereas the vast majority (>95%) of γδ T cells from naive lungs were CD25^–^, γδ T cells examined at day 7 following pneumococcal challenge showed expression of CD25. In comparison, naive lung αβ T cells were CD25^–^, and remained so following *S. pneumoniae* challenge (Fig. 4C).

Previous studies have associated γδ T cell function with the production of soluble mediators [25], including cytokines [12, 26–28]. However, *ex vivo* intracellular flow cytometry performed without exogenous stimuli did not detect expression of TNF, IFN-γ, IL-12/23 p40, IL-10 or IL-4 within γδ T cells from naive mice, or from mice at days 1, 3, 7, 10 and 14 post challenge. Quantitative real-time RT-PCR analysis of mRNA from purified, non-stimulated γδ T cells further confirmed no consistent change in cytokine mRNA accumulation (data not shown).

**Figure 5:**
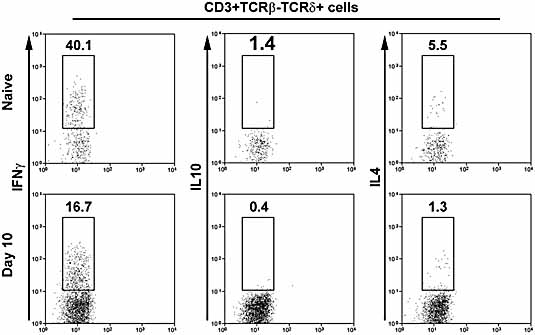
Capability of lung γδ T cells for cytokine expression. Lung cells from naive or *S. pneumoniae*-challenged mice (*n* = 5) at day 10 post challenge were stimulated *in vitro* with PMA and ionomycin in the presence of brefeldin A prior to flow cytometric analysis of cytokine expression. Gated γδ (CD3^+^TCRβ^–^TCRδ^+^) T cells stained for IFN-γ (left panels), IL-10 (centre panels) and IL-4 (right panels) are shown (all on *y* axis). Numbers indicate percentages of cytokine-positive cells, within the gates shown, among γδ T cells. Axes represent log fluorescence intensity and representative plots are shown in each case.

To determine whether the commitment of lung γδ T cells for cytokine production changed as a consequence of inflammation, lung cells were incubated in the presence of PMA and ionomycin prior to staining for cytokine expression. Given this stimulation, a high proportion of lung γδ T cells from naive mice expressed IFN-γ (35.4 ± 7.6%; Fig. 5), with few cells expressing detectable levels of IL-10 (1.3 ± 0.4%) or IL-4 (4.3 ± 1.8%). During the peak of the response, at day 10, the proportion of IFN-γ-positive γδ T cells was significantly reduced to 17.7 ± 3.4% (*p* = 0.04; *n* = 5; Fig. 5). Given the overall 30-fold increase in lung γδ T cell number, these data indicate quantitative increases in γδ T cells with IFN-γ-producing capability at this stage of the response (mean 1.8 ± 0.3 × 10^5^ IFN-γ^+^ cells) compared with naive mice (mean 0.1 ± 0.02 × 10^5^). In contrast to γδ T cells, the proportion of IFN-γ-positive CD4^+^ T cells was increased at this time point (12.5 ± 4.2%) compared with naive mice (7.1 ± 1.8%; *p* = 0.05) Among γδ T cells, the proportion of IL-10- (0.5 ± 0.2%; *p* = 0.06) and IL-4-positive (1.7 ± 0.9%; *p* = 0.12) γδ T cells also appeared to be somewhat, though not significantly, reduced. Together, these data demonstrate lung γδ T cell activation as a result of *S. pneumoniae* challenge, with elevated expression of activation markers occurring in the absence of significant increases in the proportion of cells capable of expressing common effector cytokines.

### Localized cell division of lung γδ T cells

Despite clear increases of γδ T cell numbers during inflammatory responses, the role of local expansion *versus* recruitment in this process has been implied [10, 11, 13] but not directly addressed. It was of significant interest to determine whether *S. pneumoniae*-induced γδ T cells arise through recruitment to the lung from other sites or by expansion of resident lung γδ T cells. Our flow cytometric data demonstrated no prominent increase or decrease in γδ T cell numbers in the spleen, blood and draining LN of *S. pneumoniae*-challenged mice, compared with lung (data not shown), supporting a model in which *S. pneumoniae*-induced inflammation results in expansion of a previously resident, lung-specific γδ T cell population. Therefore, lung αβ and γδ T cells were examined to determine whether these populations were undergoing expansion through division.

**Figure 6:**
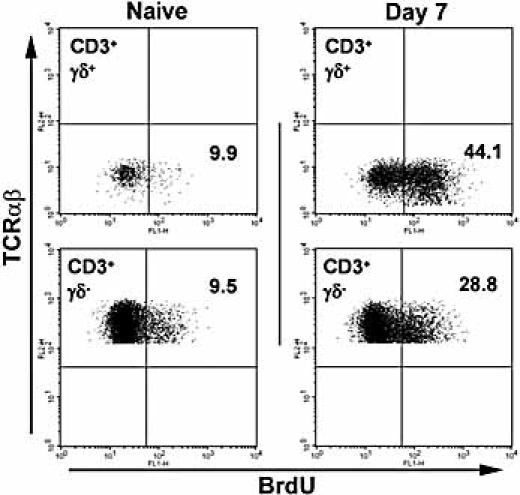
Turnover of pulmonary γδ T cells. Naive or *S. pneumoniae*-challenged mice given BrdU for 7 days to assess γδ T cell turnover. Plots show BrdU incorporation into gated γδ (upper plots) or αβ T cells (lower plots) at each time point. No positive staining was observed with isotype control mAb (data not shown).

In naive mice, similar percentages of lung γδ (12.8 ± 2.5%) and αβ T cells (9.1 ± 3.0%) incorporated BrdU over a 7-day labeling period (Fig. 6). At day 7 following *S. pneumoniae* challenge, BrdU^+^ γδ T cells were significantly increased compared with naive mice (42.2 ± 8.1%; *n* = 6, *p* <0.01). Furthermore, lung γδ T cell turnover at this time point was significantly greater (*p* <0.05) than that of lung αβ T cells (31.2 ± 4.1%; *n* = 6) (Fig. 6). Examination of γδ T cells from the spleen and draining LN of mice at day 7 post challenge did not reveal any significant increase in BrdU incorporation compared with naive controls (*p* >0.05; data not shown).

### Pulmonary γδ T cells have lung-homing capabilities

**Figure 7:**
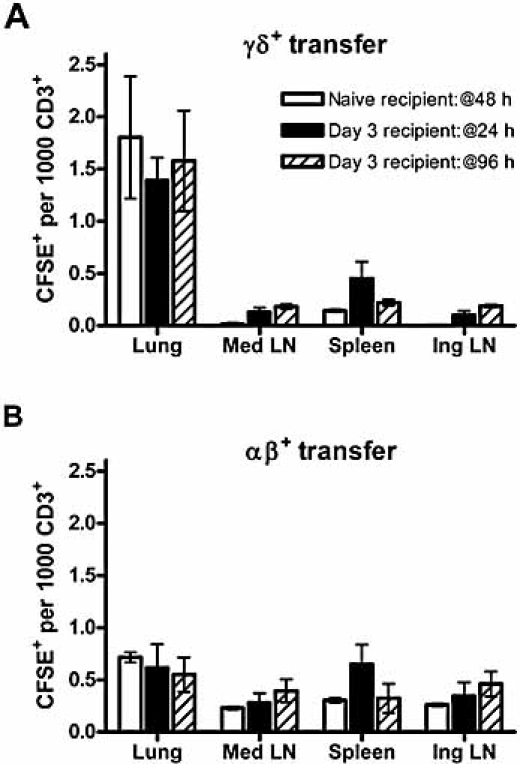
Preferential lung homing of pulmonary γδ T cells. Purified, CFSE-labeled (A) γδ T cells or (B) αβ T cells were adoptively transferred into naive recipients and analyzed 48 h later, or into recipients at day 3 post *S. pneumoniae* challenge and analyzed 24 or 96 h later. Data represent (*y* axis) mean (± 1 SD) CFSE^+^ donor cells/10^3^ CD3^+^ cells in each organ (*n* = 6 per group in two separate experiments).

Tissue-specific association of γδ T cells may be reflected in their trafficking abilities, with Vγ5^+^ cells most clearly shown to have skin-homing capability [29, 30]. To examine whether γδ or αβ T cells from *S. pneumoniae*-challenged mice were able to traffic specifically to the lung, day 7 γδ and αβ T cell populations from CD45.1^+^ donors were labeled with CFSE and transferred into naive CD45.2^+^ recipients. At 48 h post transfer, CD45.1^+^CFSE^+^ donor γδ T cells preferentially located in the lung, where they comprised a significantly greater proportion of total CD3^+^ cells than in other organs examined (Fig. 7A). Accounting for differences in CD3^+^ cells in each organ, it is estimated that 40% of the recovered CD45.1^+^CFSE^+^ donor γδ T cells were found in the lung, with approximately 50% in the spleen. In contrast, only 10% of the recovered αβ T cells were found in the lung, with more than 85% associated with the spleen. This preferential lung-specific localization also occurred following *S. pneumoniae* challenge. Thus, transfer at day 3 post *S. pneumoniae* challenge resulted in a similar distribution of CD45.1^+^CFSE^+^ donor γδ T cells at 1 or 4 days post transfer (day 4 or day 7 post challenge for recipients) (Fig. 7A). In contrast, αβ T cells purified from lungs of the same donors did not show preferential lung homing. Donor αβ T cells were detected at equivalent proportions in all organs of both naive and infected recipients that were examined (Fig. 7B). Again, accounting for differences in CD3^+^ cells in each organ at 4 days post transfer, over 50% of CD45.1^+^CFSE^+^ donor γδ T cells were found in the lung, and only 40% in the spleen. For αβ T cells, approximately 15% of the recovered cells were found in the lung, with 65% associated with the spleen. This suggests that tissue-associated inflammatory signals are not required for, but may enhance, homing of lung-derived γδ T cells.

## Discussion

Using an *S. pneumoniae*-induced model of pulmonary inflammation, this study has examined in detail the γδ T cell population involved in the ensuing immune response. A substantially increased pulmonary γδ T cell population arises as a result of pneumococcal challenge [20], and here we demonstrate the coincident involvement of multiple γδ T cell subsets. While a significant proportion of naive lung γδ T cells exhibited an activated phenotype, activation was clearly enhanced in infected mice, most notably in respect to CD25 expression. Finally, increased BrdU uptake and the preferential lung-homing capability of lung γδ T cells following pneumococcal challenge strongly suggest that local division, rather than recruitment, is responsible for this infection-induced increase in γδ T cell number.

Intranasal *S. pneumoniae* serotype 6B challenge induced a >30-fold increase in pulmonary γδ T cells, without compensatory or complementary changes within draining LN, blood or spleen. This expanded population of γδ T cells has cytotoxic activity against alveolar macrophages and lung dendritic cells, and acts to regulate these populations during resolution of inflammation [20]. However, in this and other infection models, immunoregulatory functions have been ascribed to bulk γδ T cells without detailed analysis of the receptor repertoire of the responding populations [12, 15, 16, 20, 28, 31]. Here, we have quantified Vγ1^+^ and Vγ4^+^ cells in naive lungs, confirming and extending recent immunohistochemical studies [8]. Flow cytometric analyses were unable to account for a significant proportion of lung γδ T cells in both naive and inflamed lungs, in contrast to those in draining LN, which may include pulmonary Vγ6^+^ cells [8]. While Vγ6^+^ γδ T cells have recently been identified by application of the anti-Vγ5 mAb 17D1 following staining with an antibody against the γδ T cell receptor [6, 8], this technique has not been successful in identifying Vγ6^+^ cells in our laboratory. However, PCR data support the indirect observation of pulmonary Vγ6^+^ cells [8]. While this suggests that both Vγ6^+^ and Vγ2^+^ cells are likely to comprise any remainder of the γδ T cell population, a majority of Vγ2 transcripts may be non-productive, as indicated by spectratype data [23]. Further analysis of the response did not reveal any apparent differences in the kinetics of each responding subset. This suggests that, in contrast with experimental infections such as listeriosis or schistosomiasis [32–34], there is no initial γδ T cell response associated with anti-bacterial function. In contrast, the entire response appears to correlate with the final stages of bacterial clearance and the onset of the resolution phase.

PCR analysis of naive and challenged mice also revealed lung-restricted expression of Vγ5 mRNA. While Vγ5^+^ cells are not completely restricted to the skin dendritic epithelial T cell population [17, 22, 23], pulmonary Vγ5 mRNA was unexpected. However, neither spectratype analysis nor flow cytometry, in agreement with recent data [8], indicated the presence of any currently defined Vγ5^+^ population. Non-productive Vγ5 mRNA may be erroneously expressed by, for example, Vγ6^+^ cells, as suggested by previous studies [35–37]. Therefore, lung γδ T cells expressing functional Vγ5 TCR appear to have been excluded.

Vγ1^+^ and Vγ4^+^ cells were significantly increased following *S. pneumoniae* challenge. Pulmonary Vγ1^+^ and Vγ4^+^ cells have previously been shown to respond to both mycobacteria [13] and influenza [11]. While γδ T cell cytokine production is observed in both models, only mycobacterial challenge induces γδ T cell cytotoxicity [12, 13, 28]. In contrast, pneumococcal challenge induces resolution-associated cytotoxic activity [20] without coincident *ex vivo* cytokine expression. Stimulation of γδ T cells with PMA and ionomycin revealed no overall change in the capacity of these cells to produce specific cytokines following challenge. While these data suggest increased numbers of γδ T cells with cytokine-producing potential during the inflammatory response, any role for γδ T cell-derived cytokines in the current model remains to be defined.

It remains unclear whether Vγ1^+^ and Vγ4^+^ populations recognizing similar targets are expanded in these disparate contexts, suggestive of functional heterogeneity dependent on an inflammatory environment rather than the ligand encountered. Alternatively, each inflammatory situation may activate distinct populations of Vγ1 and Vγ4 cells, each with specific functions. Despite this first quantification of pulmonary γδ T cell subsets during inflammation, a majority of responding γδ T cells remain undefined by direct observation. Nevertheless, by implication from PCR analyses together with flow cytometric data, *S. pneumoniae*-induced inflammation clearly induces substantial quantitative increases in multiple γδ T cell subsets.

The majority of γδ T cells from naive lungs constitutively expressed the activation-associated markers CD44 and CD69. This apparently high level of constitutively activated pulmonary γδ T cells fits within a model of γδ T cell population maturation in naive animals [38]. Phenotypically activated, ‘naive’ γδ T cells also occur in murine vaginal [39, 40] and intestinal epithelium [41], but not the spleen of the same uninfected mice, or in recent thymic emigrants [38]. These data suggest that a mucosal location may closely associate with an activated status in apparently ‘naive’ hosts, although the relationship between activation and homeostatic function remains to be investigated. *S. pneumoniae* challenge further activated the multiple responding γδ T cell subsets in a surprisingly uniform fashion. The activation response is not restricted to, for example, the Vγ1 or Vγ4 subset, as for Vγ6 in both *Listeria* [6] and pulmonary fibrosis [10] models. In other systems, γδ T cells may up-regulate surface markers following recognition of Toll-like receptor ligands [42], or as a result of TNF activity [43], although the mechanisms driving activation of γδ T cells both in naive lungs and following *in vivo* pathogen challenge remain unknown.

Given the tissue-specific nature of certain γδ T cell subsets, it may be assumed that increases in lung γδ T cells arise from local expansion. While data concerning canonical Vγ subsets, including Vγ6 in the lung [10], strongly support this conclusion, other γδ T cell subsets have widespread distributions. Thus, while increased Vγ1 and Vγ4 numbers may represent local expansion, these cells may feasibly migrate to the lung from draining LN, spleen and other lymphoid tissues. The current data are the first to directly examine this alternative, and indicate that local expansion is the primary source of increased numbers during a polyclonal γδ T cell response. Following *S. pneumoniae* challenge, cell division among γδ T cells is significantly increased in the lung, but not in draining LN or spleen, and cycling of lung γδ T cells has been observed in response to influenza infection [44]. The inflammatory environment of the challenged lung may drive both activation and expansion of resident γδ T cells. The lack of significant γδ T cell division, or their increase or loss in other tissues and blood, supports this model. However, any potential contribution of thymic emigrant cells, while of a non-activated phenotype in naive animals [38], remains to be determined under inflammatory conditions. While the proportion of cycling (BrdU^+^) lung αβ T cells was also increased following challenge, this is not accompanied by substantial increases in their number. In contrast to the apparent local expansion of γδ T cells, it is more likely that αβ T cells expand in draining LN and home back to lung tissues. The absence of substantial quantitative changes suggests that the current model of rapidly resolving inflammation does not facilitate the prolonged retention or recruitment of αβ T cells within the lung.

γδ T cells from lungs of mice challenged 7 days previously with *S. pneumoniae* exhibit a strong preference in homing to the lung, regardless of the inflammation state of the tissue. This inflammation independence contrasts with the homing phenotype suggested from human *in vitro* studies [45], and may reflect the tissue, rather than blood, origin of the γδ T cells. That lung-derived αβ T cells from the same individuals did not exhibit this preference is further evidence of the tissue-specific nature of both the lung γδ T cell response and of γδ T cells in general. Two important considerations remain under examination with regard to homing capabilities. First, does each lung γδ T cell subset have equal homing capacity? Further studies will examine whether lung homing is most associated with the canonical lung Vγ6 subset. Second, is γδ T cell homing to the lung restricted to cells that originate in the lung, or is it an inflammation-inducible property of γδ T cells in other tissues? To address this latter point, we attempted to purify activated γδ T cells from the spleen of *Listeria*-infected mice for transfer studies. However, it was not possible to purify sufficient splenic γδ T cells due to their strong adherence to activated splenic macrophages in this model ([46] and our unpublished data).

*In vitro* studies of human γδ T cells suggest that these cells have LN-homing capabilities once activated [47], while others may acquire a tissue-homing phenotype [45]. It is possible that lung γδ T cells may be ‘imprinted’ for lung homing either during development or activation, as occurs for αβ T cells in the intestinal mucosa [48]. The mechanisms governing lung-specific γδ T cell homing are the subject of ongoing study, although expression of the commonly mucosal lymphocyte-associated integrin CD103 by pulmonary γδ T cells has not been observed (our unpublished observations).

Together these studies provide further evidence for the tissue- and context-specific nature of γδ T cell responses. However, they also serve to highlight the paucity of knowledge concerning γδ T cell behavior and its regulation. By further examination of the activation processes and tissue-specific regulatory mechanisms to which γδ T cells are subject, a much better understanding of this important, immunoregulatory T cell population will emerge.

## Materials and methods

### Mice and *S. pneumoniae* infection

C57BL/6 (B6.CD45.2) and B6.CD45.1 mice were bred and housed under barrier conditions at LSHTM and the University of York, and supplied with food and water *ad libitum*. During *in vivo* labeling experiments, drinking water was supplemented with BrdU (Sigma) at 0.8 mg/mL, freshly prepared daily. Mice were infected intranasally with approximately 10^8^ CFU of *S. pneumoniae* serotype 6B as described [18, 19]. Animal experimentation was performed with LSHTM and the University of York Animal Procedures Ethics Committee and UK Home Office approval.

### Tissue preparation

Whole organs were collagenase-digested to single-cell suspensions as described [18]. Viable cell counts were determined by Trypan blue exclusion.

### Flow cytometry and cell sorting

Flow cytometry was carried out as described [18], using the following antibody clones (all BD Pharmingen): GL3 (TCRδ), 536 (Vγ5), UC310A6 (Vγ4), 8F4H7B7 (Vδ6.3), 145.2C11 (CD3), H57–597 (TCRβ), GK1.5 (CD4), 53–6.7 (CD8α), RA3.6B2 (B220), PC61 (CD25), IM7 (CD44), H1.2F3 (CD69), A20 (CD45.1), XT22 (TNF), C15.6 (IL-12 p40/70, IL-23), JES5–16E3 (IL-10), XMG1.2 (IFN-γ), 11B11 (IL-4), and appropriate isotype controls. AlexaFluor488 (Molecular Probes)-conjugated F(ab’)_2_ fragments of GL3, 2.11 (Vγ1) and UC310A6 were prepared in-house (hybridomas generously provided by P. Pereira, Institut Pasteur, Paris, France). BrdU was detected using FITC-conjugated anti-BrdU antibody (clone B44; BD Pharmingen), according to standard protocols [38]. Intracellular cytokine staining followed a 4-h incubation with brefeldin A alone (Sigma) as described [49], or in the presence of PMA and ionomycin. All samples were treated with anti-FcRII/III mAb (clone 2.4G2) prior to specific staining. Samples were acquired on a FACSCalibur flow cytometer and analyzed with CellQuest Pro software (both Becton Dickinson, Oxford, UK), or on a Cyan flow cytometer and analyzed with Summit v4.1 software (both DakoCytomation).

For flow cytometric sorting, whole lung preparations were enriched for T cells using biotinylated anti-CD3 antibody, anti-biotin beads and MACS sorting (Miltenyi Biotech, Germany). Subsequent purification of γδ and αβ T cells was carried out on a MoFlo cell sorter (DakoCytomation). Purity of sorted populations was confirmed by flow cytometry as >95% in each case.

### Adoptive transfer

Sorted γδ or αβ T cells were labeled by incubation in 5 µM CFSE for 10 min at room temperature, washed extensively and resuspended in PBS. Mice received 10^6^ donor cells intravenously.

### PCR and spectratype analysis

RT-PCR analysis of Vγ chain expression, and subsequent spectratyping of PCR products, was carried out on mRNA extracted from whole organ preparations, or from sorted cell populations derived from pooled organs of three to six individuals, as described [23].

### Statistical analysis

Where indicated, data were compared using Student's two-tailed *t*-test.
